# Fractal dimension of coastline of Australia

**DOI:** 10.1038/s41598-021-85405-0

**Published:** 2021-03-18

**Authors:** Akhlaq Husain, Jaideep Reddy, Deepika Bisht, Mohammad Sajid

**Affiliations:** 1 Department of Applied Sciences, BML Munjal University, Gurgaon, Haryana 122413 India; 2 School of Engineering and Technology, BML Munjal University, Gurgaon, Haryana 122413 India; 3 Department of Mechanical Engineering, College of Engineering, Qassim University, Buraidah, Al Qassim 51452 Saudi Arabia

**Keywords:** Geomorphology, Geophysics, Computational science, Computer science, Software

## Abstract

Coastlines are irregular in nature having (random) fractal geometry and are formed by various natural activities. Fractal dimension is a measure of degree of geometric irregularity present in the coastline. A novel multicore parallel processing algorithm is presented to calculate the fractal dimension of coastline of Australia. The reliability of the coastline length of Australia is addressed by recovering the power law from our computational results. For simulations, the algorithm is implemented on a parallel computer for multi-core processing using the QGIS software, *R*-programming language and Python codes.

## Introduction

From clouds to mountains, snowflakes to river networks, broccoli to blood vessels, fractals can be noticed everywhere in nature. The application of fractals can be seen in fractal antennas, digital imaging, computer graphics, computational geometry, geology and many other fields. Man made fractals include the Cantor set, Sierpinski triangle, and the Mandelbrot set etc.

Coastlines (the boundary between land and water) and other natural boundaries have been a subject of human fascination since long. Their construction is random as compared to deterministic fractals such as the Mandelbrot set (which is formed through repeated iterations of a simple mathematical equation). The irregularities and variations in coastlines have defied attempts of characterization using methods which are based on Euclidean geometry. Measuring the length of smooth curves is a simple process of successive approximations by line segments. For more accurate measurements, smaller segments can be used. As the segment size approaches zero, the sum of the lengths of the segments will approach the length of the curve. However, this naive application of Euclidean geometry to natural curves fails because the limit may not exist. There are several possibilities for this failure such as sloppy length measurements, use of different data sets by different researchers (some of which may be erroneous), and use of different measurement methods, leading to differences in measured lengths etc.

The variation in the lengths of coastlines at different scales intrigued the scientist Lewis Fry Richardson in the 1920s. He examined the coastlines of several countries including Great Britain, South Africa, and Australia as well as the border of Spain and Portugal^35^. He chose to measure a coastline length by walking a divider of a specific length along the coastline to see how many dividers were needed to cover the entire coastline and calculated the length of the coastline by multiplying the number of dividers by the length of each divider.

There are various numbers, associated with fractals, which provide an objective means for comparing fractals. They are generally referred to as fractal dimensions. These numbers attempt to quantify how densely the fractal occupies the space in which it lies. Many variations of fractal dimension have been defined, and for some sets, these different dimensions yield different values. Fractal dimension which itself is a class of different dimensions (such as the Hausdorff dimension, similarity dimension, box-counting dimension and the divider dimension) which are all equal for exactly self-similar fractals like the Koch curve. We refer the reader to the classical text books by Barnsley^1^, Falconer^5^, and the book by Frame et al.^13^ for a comprehensive study of various types of fractal dimensions and methods to calculate these. The recent book by Fernández-Martínez et al.^11^ is a good reference from both theoretical and applied viewpoints which is focused on the calculation of the fractal dimension of an object in more general settings.

The inspiration to calculate the fractal dimension of coastlines came with the landmark paper of Mandelbrot “*How long is the coast of Britain? Statistical Similarity and Fractal Dimension*”^28^. The answer depends on how closely you look at it, or how long is your measuring stick. Mandelbrot’s research^28–30^ showed that the fractal dimension of a coastline is constant, whereas the length varies in accordance with different measurement scales.

After the pioneering work of Mandelbrot, the fractal dimensions of different coastlines have been calculated by many researchers, including Mandelbrot^28^, Goodchild^17^, Kappraff^22^, Philips (1986), Feder^6^, Longley and Batty^26^, Carr and Benzer^2^, Jay and Xia^20^, Paar et al.^33^, Jiang and Plotnick^21^, Zhu^42^, Ma et al.^27^ and others. Applications of fractal dimension have also been investigated by a number of researchers and we refer to the papers by Cosandy (2002), Dimri^3^, Fernández-Martínez et al.^10^, Gonzato^16^, Hayward et al.^19^, Khoury and Wenger^23^, Li et al.^25^, Wu et al.^40^, Zhang et al. (2002) and the books by Korvin^24^, Turcott^38^ for applications of fractal dimension in many other exciting fields of research. We also refer to the interesting paper by Fernández-Martínez et al.^7^ for models of fractal dimension calculation in non-Euclidean contexts. In addition, use of GIS tools (e.g. ArcGIS, QGIS etc.) for calculating fractal dimensions have proven to be generous and a number of researchers have used these tools for estimating fractal dimension.

In this article, we calculate the fractal dimension of coastline of Australia using the box-counting method. For simulations we have designed a novel, scalable, multicore parallel processing algorithm and implemented the algorithm on a parallel computer using QGIS software (Pi-version) and Python codes to speedup the processing time and efficiency of computations. We also discuss the reliability of the coastline length of Australia, which is in use at present, by recovering the inverse power law in computing the fractal dimension using the data from simulations. The only available results for fractal dimension of Australia are from the earliest works by Mandelbrot^28^ and Richardson^35^ using the divider method. The algorithm can be used to compute fractal dimension of any coastline and other natural objects such as mountains, rivers, glaciers etc. This makes the study and the algorithm important and exciting.

This paper appears to be the first to obtain box-counting dimension of Australia and also the first to introduce a multi-core parallel processing algorithm for computing box-counting dimension in the context of coastlines and otherwise too.

## Fractal dimension: a brief review

Fractals model broken, jagged, complex, wiggly and rough shapes. But some shapes are rougher and more complex (e.g. coastlines) than others. The fractal dimension is used to quantify this roughness and complexity. The higher the dimension, the higher the roughness, the higher the complexity.

The earliest known measure of roughness of an object is the Hausdorff dimension (also known as Hausdorff-Besicovitch dimension) introduced by Felix Hausdorff in 1918 even before the term fractal was coined, and much of the early work on the subject was done by A.S. Besicovitch. It is now considered one of the fractal dimensions because it has been useful in describing the properties of many fractal sets. For a complete treatment of Hausdorff dimension we refer to the book by Edgar^4^. A computational approach to calculate the Hausdorff dimension of compact Euclidean subsets was given by Fernández-Martínez and Sánchez-Granero^9^ and we refer to the recent work by Fernández-Martínez et al.^12^ for calculation of the Hausdorff dimension in higher dimensional Euclidean spaces.

**Figure 1:**
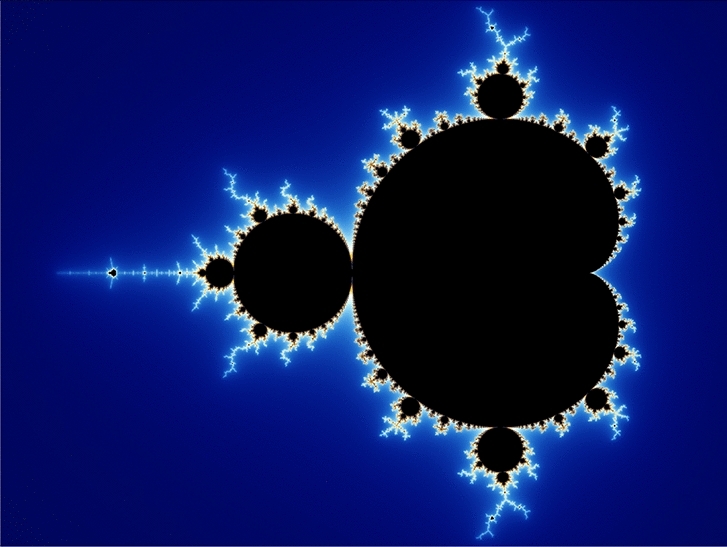
The Mandelbrot set.

About his exploratory work on the fractal dimension of coastlines^28^, B.B. Mandelbrot quoted in his famous book *The Fractal Geometry of Nature* (1982), “*Clouds are not spheres, mountains are not cones, coastlines are not circles, and bark is not smooth, nor does lightning travel in a straight line*”. Mandelbrot discovered and plotted one of the most interesting and popular fractal known as the Mandelbrot set (see Fig. 1) at IBM on March 1, 1980.

Shishikura^36^ proved the intriguing conjecture by Mandelbrot^32^ which states that the Hausdorff dimension of the boundary of the Mandelbrot set is 2. Shishikura’s proof was based on the concept of bifurcation of parabolic periodic points.

Let us begin by introducing the formal definition of fractal dimension.

### Definition 1

Let (*X*, *d*) be a metric space and *A* be a compact subset of *X*. Let $\epsilon >0$ (a small number) be given and define

$$\begin{aligned} N_\epsilon (A) = \text{ minimum } \text{ number } \text{ of } \text{ closed } \text{ balls } \text{ which } \text{ covers } \text{ A } \end{aligned}$$

i.e. $A\subset \displaystyle {\bigcup _{i=1}^{n}}{B_{\epsilon }(x_i)}$, where $B_{\epsilon }\left( x_i\right)$ is a ball of radius $\epsilon$ centered at $x_i$. We say that *A* has fractal dimension *D* if

1 $$\begin{aligned} N_\epsilon \left( A\right) \approx C\epsilon ^{-D} \end{aligned}$$

where $C>0$ is a constant.

In the above definition $\approx$ means that if $f(\epsilon ), g(\epsilon )$ be two functions of $\epsilon$ then $f\approx g$ if and only if $\displaystyle {\lim _{\epsilon \rightarrow 0}}\frac{f(\epsilon )}{g(\epsilon )}=1$. From Eq. (1), we obtain

2 $$\begin{aligned} D=\lim _{\epsilon \rightarrow 0}\frac{\log \left( N_\epsilon (A)\right) }{\log \left( \frac{1}{\epsilon }\right) } \end{aligned}$$

provided the limit exists.

We recall two important results from Barnsley^1^. The first result gives existence of fractal dimension and the second result replaces the continuous variable $\epsilon$ in Eq. (2) by a discrete variable to simplify the process of computations.

### Theorem 1

(Theorem 2.1, Chapter 4) *Let* *m* *be a positive integer. Consider the space* $\left( \mathbb {R}^m, Euclidean\right)$*. Then* *D*(*A*) *exists for all* $A\in H(\mathbb {R}^m)$*. Here,* $H(\mathbb {R}^m)$ *is the set of all* *non-empty compact subsets of* $\mathbb {R}^m$.

In particular, the theorem guarantees that the fractal dimension exists for every coastline since coastlines are closed and bounded (compact) subsets of $\mathbb {R}^2$.

### Theorem 2

(Theorem 1.1, Chapter 4) *Let* *A* *be a compact subset of a metric space* (*X*, *d*)*. Let* $\epsilon _n=Cr^n$ *for each real number* $0<r<1$ *and* $C>0$*. Then* *A* *has fractal dimension* *D* *given by*

3 $$\begin{aligned} D=\lim _{n\rightarrow \infty }\left\{ \frac{\log \left( N_{\epsilon _n}(A)\right) }{\log \left( \frac{1}{\epsilon _n}\right) }\right\} . \end{aligned}$$

The divider (or compass) dimension is another fractal dimension which is more relevant for coastlines. It is defined for all curves, and a natural choice for characterization of coastlines. The divider dimension examines both the relationship between scaling size and the length of the curve and the relationship between scaling size and the number of segments at the size that is needed to cover the curve as follows.

A pair of dividers is opened to a step length $\epsilon$ and is walked along the curve until the end is reached after $N(\epsilon )$ steps. The length of the curve is measured as $L(\epsilon )=\epsilon \cdot N(\epsilon )$. When $\epsilon$ is large, this method skips many irregularities along the curve, but as $\epsilon$ decreases, the finer features of the curve are also included, and the overall length of the curve increases. Richardson was the first to use the divider method in his work on coastlines^35^ and showed that this behavior is a power law with

4 $$\begin{aligned} L(\epsilon ) = k\times \epsilon ^\alpha \, \text{ where }\, k, \alpha \, \text{ are } \text{ constants }. \end{aligned}$$

Mandelbrot^28,31^ later discovered with exactly self-similar fractals that $\alpha =1-D$ where *D* is the similarity dimension. Thus, $L(\epsilon )=k\times \epsilon ^{1-D}$. Upon taking the logarithm of both sides,

5 $$\begin{aligned} \log L(\epsilon ) = \log k + (1-D) \log \epsilon . \end{aligned}$$

A plot of $\log (\epsilon )$ on the $x-$axis vs. $\log L(\epsilon )$ on the $y-$axis results in a line which has approximate slope $1-D$. The double logarithmic plots of coastline length versus the step length are called *Richardson plots*. Thus, power law behavior results in linearity on a double logarithmic plot (also called as $\log -\log$ plot). The resulting value of *D* is called the *divider dimension*.

Of course, the data from length measurements of natural fractals is not exactly linear, but the approximation is often good enough to use the least squares or regression method for a close linear fit. Richardson used this technique when he observed the power-law behavior with the coastline of Great Britain.

It follows that $\epsilon \times N(\epsilon )=k\times \epsilon ^{1-D}$ so that $N(\epsilon )=k\times \epsilon ^{-D}$. This gives

6 $$\begin{aligned} \log N(\epsilon ) = \log k- D \log \epsilon . \end{aligned}$$

When using a double logarithmic plot, the slope of the resulting line will be approximately $-D$.

The reported lengths of the border between two countries were often claimed to be different by the countries involved. Richardson^35^ described the regularity between the length of coastline boundaries and scale size by observing that the length of coastlines increases rapidly as the length of line segments decreases, instead of approaching a limiting value (as it does for a smooth curve) it appears to grow without bounds.

To understand this growth, Richardson plotted $\log (L)$ vs. $\log (\epsilon )$, obtaining points approximately along straight lines with slopes given in Table 1. The slope of the line was found $1-D$ (by Mandelbrot) which on solving for *D*, gives the values in Table 1. For Great Britain, $1-D=-0.25$, so that the fractal dimension of Great Britain is 1.25. For the coastline of South Africa $D=1.02$. This makes sense because the coastline is nearly a regular Euclidean object, i.e. a line, which has dimension 1. Mandelbrot^28^ interpreted this value of *D* as the fractal dimension.

**Table 1:** Slopes for coastlines computed by Richardson^35^ and fractal dimension (*D*) computed by Mandelbrot^28^.

S. No.	Coastline	Slope	Fractal Dimension (*D*)	Reference Figure
1	West coast of Britain	$-0.25$	1.25	Figure 2a
2	Land frontier of Germany	$-0.15$	1.15	Figure 2b
3	Land frontier of Portugal	$-0.14$	1.14	Figure 2c
4	Coastline of Australia	$-0.13$	1.13	Figure 2d
5	Coast of South Africa	$-0.02$	1.02	Figure 2e

A major difficulty with the divider dimension is ambiguity in how to cover the curve with segments of a particular scaling factor. This problem is mainly due to multiple forward intersections at a particular stepsize. An additional problem is how to compensate for the leftover portion of the curve which has length less than $\epsilon$. When $\epsilon$ is small, so is the error, but larger values of $\epsilon$ leads to greater errors.

To overcome these difficulties the box-counting dimension $D_b$ is used. $D_b$ is also an exponent in a power law relation just like fractal dimension $D$. This is one of the most commonly used dimensions because of its simplicity for machines and it can be applied to any object in space. It is a simplification of the Hausdorff dimension, and for many fractals like Koch curve etc., the box-counting dimension will be equal to the other fractal dimensions.

There are several different versions of the box-counting dimension, and the one to be discussed here relates to curves. A grid size $\epsilon$ is chosen and a grid is drawn on the given curve with boxes of size $\epsilon \times \epsilon$. Then, the number of boxes $N(\epsilon )$ which contain a portion of the curve are counted. $N(\epsilon )$ will usually increase as $\epsilon$ decreases. For fractals, a plot of $N(\epsilon )$ vs. $\epsilon$ on a double logarithmic scale is typically linear with slope $-D_b$. The number $D_b$ is called the *box-counting dimension*. The scaling hypothesis is that $N(\epsilon )$ is related to $\epsilon$ by a power law,

$$\begin{aligned} N\left( \epsilon \right) = k\cdot \left( 1/\epsilon \right) ^{D_b}. \end{aligned}$$

Depending on the information available on $N(\epsilon )$, the value of $D_b$ can be calculated in two ways. For some objects, an explicit formula for $N(\epsilon )$ can be found and in that case $D_b$ is computed by taking a limit of the appropriate expression (see Theorem 3 below). For physical fractals and random fractals (such as coastlines), an exact formula for $N(\epsilon )$ may not be available. In such cases, $D_b$ is computed by measuring the slope of a graph. The later is being used for computing fractal dimension of coastlines.

As a particular case of Theorem 2, we have the box-counting theorem^1^,

### Theorem 3

(The Box Counting Theorem) *Let* *A* *be a closed and bounded subset of* $\mathbb {R}^2(\mathbb {R}^3)$*. Cover* $\mathbb {R}^2(\mathbb {R}^3)$ *by square boxes of side length* $\frac{1}{2^n}$*. Let* $N_n(A)$ *be the number of boxes that intersect* *A. Then the box counting dimension of* *A* *is given by*

7 $$\begin{aligned} D_b(A)=\lim _{n\rightarrow \infty }\left\{ \frac{\log (N_n(A))}{\log (2^n)}\right\} . \end{aligned}$$

A novel theory that generalizes the classical box-counting dimension and fractal dimension to any space equipped with a fractal structure was given by Fernández-Martínez and Sánchez-Granero^8^ which include Theorem 3 as a particular case.

**Figure 2:**
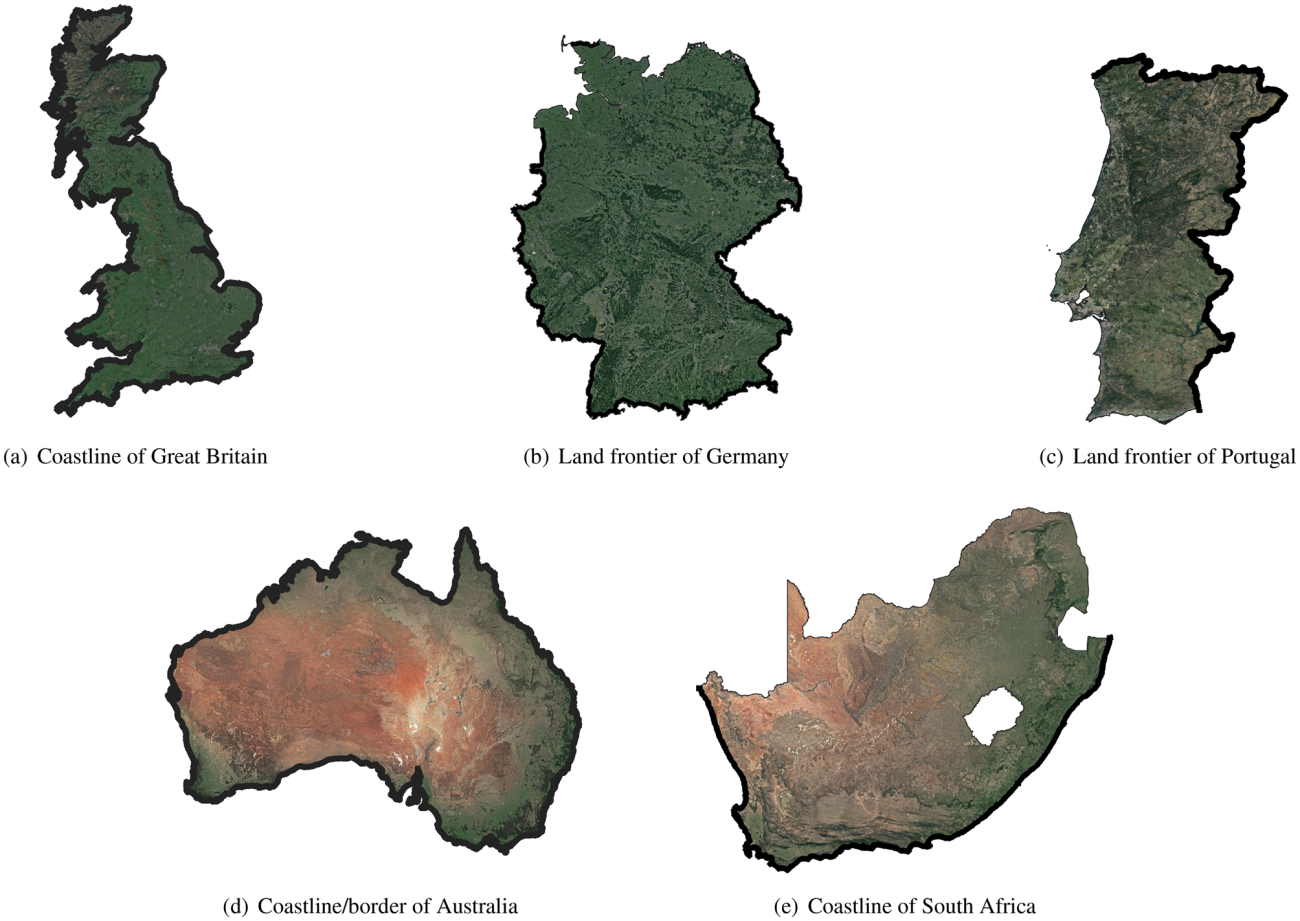
Some coastlines and borders created using QGIS, Pi-version (available at https://qgis.org/downloads/).

Coastlines, like many other geological features, exhibit similar kinds of structure over a range of scales. That is, they are scale invariant, at least for some range. This is expected because the forces that sculpt coastlines such as wind, tides or erosion operate in approximately the same way over a wide range of scales. Figure 2 shows some coastlines and borders (created using the QGIS (Pi-version) by obtaining the shapefiles from GADM and imposing the respective country polygons on the Google satellite map and extracting the intersection for each image) which have been focus of interest over last several decades by many authors (see also Table 1 and Table 2). For clarity, we have marked the border/coastline of interest with thick black color.

The fractal dimension of a coastline is a characteristic parameter to describe the irregular extents of coastlines, and it has a value between 1 and 2. Coastlines with higher fractal dimension are more complex and irregular while those with lower dimension are more smoother. Several methods have been proposed in the literature to estimate the fractal dimension of coastlines, including the divider method, the box-counting method, the stochastic noise method etc. These methods have been exploited by a number of researchers to calculate the fractal dimensions of coastlines of different regions (See Table 1 and also Table 2 for a partial list of available results).

Some researchers have discussed the scaling regions of coastlines (see Ma et. al.^27^ and references therein). Scaling region refers to the curved segment of the $\log N-\log \epsilon$ curve on removal of which the line

8 $$\begin{aligned} \log (N)=-D \log (\epsilon )+ C \end{aligned}$$

fits automatically to the computed data. Here, *C* is the intercept and *D* is the fractal dimension. Scaling region helps to decide the correct value of the measurement scale to be used, and hence more accurate length and dimension. The fractal dimension is inaccurate if the data outside the scaling regions are involved in the calculations^3^. Thus, the study of the measurement scale of the coastline’s length is required to verify whether it is within the scaling region.

**Table 2:** Chronological calculations of fractal dimension (*D*) for various coastlines. “−”denotes no data available.

S. No.	Coastline	Divider dimension	Box-counting dimension	Reference(s)
1	West Coast, Great Britain	1.25	−	Mandelbrot^28^
2	Australia	1.13	−	Mandelbrot^28^
3	Coastline of South Africa	1.02	−	Mandelbrot^28^
4	Delware Bay, New Jersey	1.46	−	Philips (1986)
5	South Coast, Norway	−	1.52	Feder^6^
6	East Shore, Gulf of California	1.02	−	Carr and Benzer^2^
7	West Shore, Gulf of California	1.03	−	Carr and Benzer^2^
8	Cres Island, Croatia	−	1.118	Paar et al.^33^
9	China	1.16	−	Zhu et al.^42^
10	China	1.195	−	Su et al.^37^
11	Shangdong and Tianjin, China	−	1.1383	Xu et al.^41^
12	Continental coastline of China	1.2004	1.0929	Ma et al.^27^

## Algorithm & implementation

We now describe our algorithm and its implementation using the box-counting method. For the purpose of map selections we have used GADM maps^14^. GADM provides database and high spatial resolution maps in export formats, including shapefiles that are used in most common GIS applications. In all our simulations, we have used recently released Pi-version of the QGIS^34^. QGIS is an open-source geographic information system (GIS) software that supports viewing, editing, and analysis of geospatial data.

First we obtain a high spatial resolution coastline map from the GADM database as a single ‘multi-polygon’ (a single feature) having a longitude/latitude coordinate reference system. The map is then transformed to a planar coordinate system using the “Australian National Grid” coordinate reference system which make use of the Transverse Mercator projection as a map projection system. This transforms the coordinates of the map from longitude latitude to the chosen coordinate reference system. Since we do not include the islands in our study therefore, we need to separate (disaggregate) different polygons. For this, we use our ‘*R*’-program which converts the entire map into a set of features out of which we select the largest (that corresponds to the mainland).

**Figure 3:**
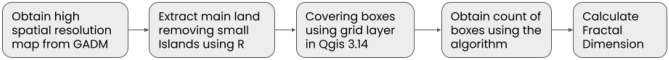
Flow chart of the box-counting method in our analysis.

Now we superimpose a grid of boxes of a given size (input) on the coastline map and the number of boxes that intersect the coastline are stored. This is accomplished using ‘grid layer’ in the QGIS. The process in then repeated with boxes of different scales. This is done using a Python program. The slope of the Richardson plot between the box-count and the box-size gives the fractal dimension of the coastline. Flowchart for the process is shown in Fig. 3.

Despite being a powerful method for estimating the fractal dimension, the box counting method is computationally slow due to increasing problem size with repeated subdivisions of the object at different sizes. To circumvent this problem and to speedup the computations for large size images (e.g. in coastlines) or large number of images (e.g. in medical imaging) one needs to design methods with reduced complexity both at algorithm and implementation level. The Higuchi dimension (Ahammer, 2011) and correlation fractal dimension (Zhang et al., 2002) are computationally faster than the box counting method. However, when with increasing problem size, new computational algorithm involving parallel or multicore processing are required.

Parallel implementations are based upon mapping the group of boxes to different compute processors and combine the results to get the total number of boxes intersecting a coastline or shape. We refer to the works of Mukukdan (2015) and Ruiz and Jimènez (2016) for significant efforts in this direction with GPU, OpenCL implementations for computing the fractal dimension of self-similar fractals.

On a typical landscape attributes like trees, roads, houses, rivers etc. represent a feature. These features can be represented by vectors in a GIS application containing text or numerical information that describe the features.

The algorithm presented in this section is based on a sub-parallel multiprocessing approach. Let us assume that *N* multi-processor nodes are available. The array of grid cells or features (*G*) is divided by *N* and each part containing $\frac{G}{N}$ features is run on each node. Now, if there are $C_n$ cores on each node then each core on a particular node search for $\frac{G}{N\cdot C_n}$ features if any of them intersect with the coastline vector.

If the time taken to process *G* features using a single node with $C_n$ cores is *T* then with each core running in parallel it will take $\frac{T}{C_n}$ time to compute $\frac{G}{C_n}$ features or grid cells. Using *N* nodes, the computational time will be reduced to $\frac{T}{C_n\cdot N}$. Therefore, the percentage gain in speed of computation is

9 $$\begin{aligned} \text{ Percentage } \text{ gain }=\frac{T-\frac{T}{N\cdot C_n}}{T}\times 100 =\frac{N\cdot C_n-1}{N\cdot C_n}\times 100\%. \end{aligned}$$

Our approach is embarrassingly parallel since there is no dependency or need for communication between different processors in our parallel tasks. The high performance cluster used in computations consists of 6 compute nodes (1 master and 5 slave). Each node is a dual core Intel Xeon 4116 processor with 20 cores on each processor powered by Intel Cluster Management Studio. The available memory on each node is 96GB along with 24TB IB enabled storage.

**Figure 4:**
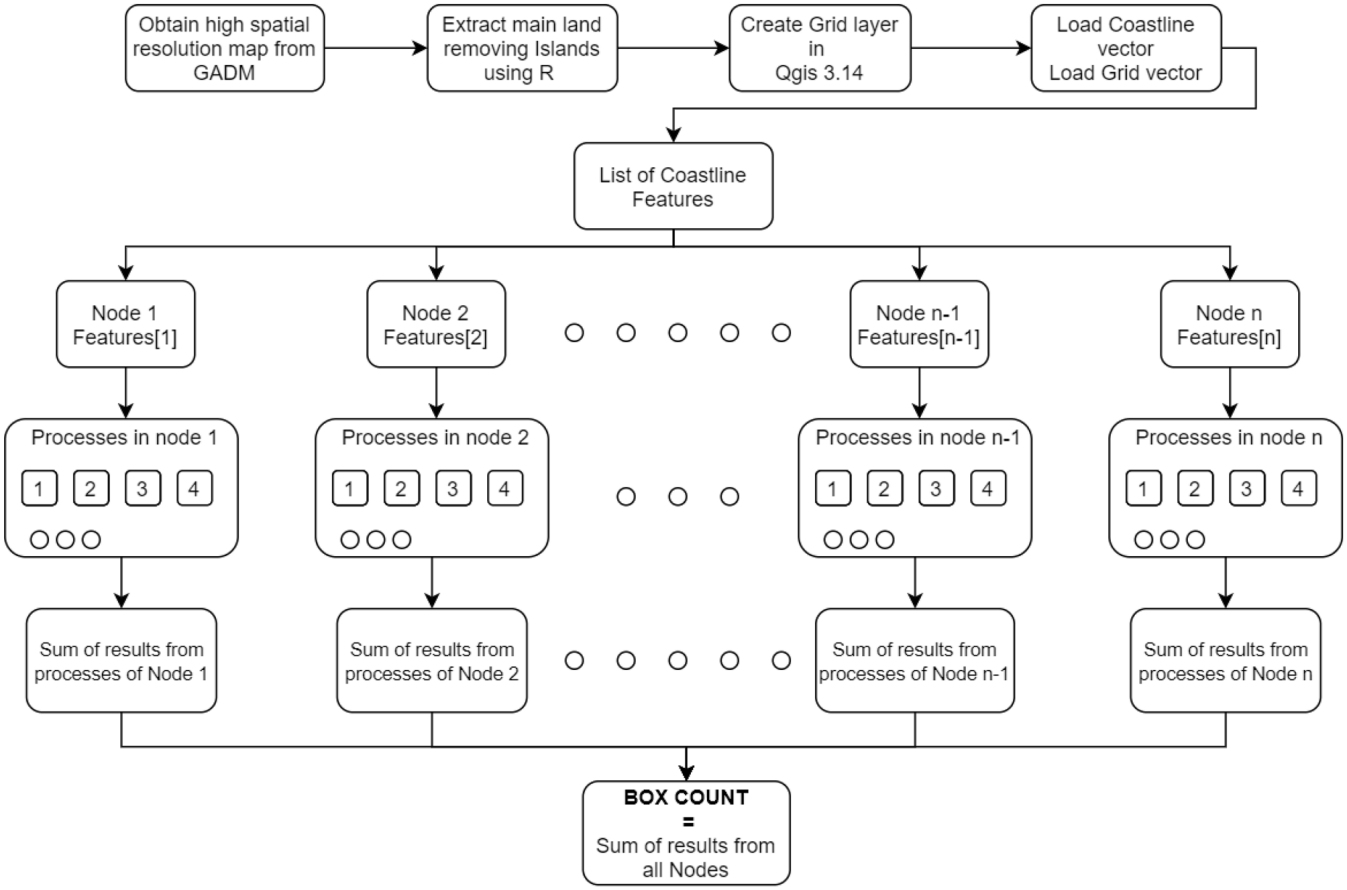
Flow chart of the parallel implementation.

Figure 4 displays flow chart of the proposed parallel implementation. Multiprocessing library is used to create processes and retrieve the results from all the processes. To collect results from all processes we need a shared storage, multiprocessing module provides Array (a C type array allocated from shared memory) and Value objects to share data between processes. We initialize a Multiprocessing Array and pass it as an argument to the function during the creation of process. Along with array we also send the index of the processes to store result in that index without disturbing the values of other processes. The join() function is used to wait till all the processes are completed. Then we add the elements of multiprocessing array to get the box-count.

Plots for all computational results are created using Veusz software package^39^, which is a scientific data plotting and graphing program written in Python, PyQt and NumPy developed by Jeremy Sanders at the Max Planck Institute for Extraterrestrial Physics (MPE).

## Results

Matthew Flinders quoted Australia as the largest island in the world on completing its first circumnavigation in 1803. Later, the length of the coastline of Australia was found to be significantly different by independent official sources. For example, the 1978 Year Book of Australia quoted it as 36735 km, the Australian Encyclopedia gives the length as 19658 km while the Australian handbook quoted the length as 19320 km.

To obtain a dependable length estimate, Galloway et al.^15^ covered the Australian coast using 162 maps from the NATMAP digital maps and measured the coastline length using a divider. The total length of the coastline including the length of coast of all the islands was found to be 47070 km. The current coastline length as reported^18^ is 59681 km (mainland coastline 35821 km and island coastline 23860 km). Looking at these numbers one would immediately wonder which one is correct? The answer is: all of them! Each source used a ruler with different size.

Our analysis will consider the discrepancy in the coastline length by increasing the number of measurement scales and identifying the scaling region. The length of the coastline is calculated to see if it lies within the scaling region for reliable length. This makes the study important and useful because it allows to reveal the fractal characteristics of the coastline and also helps to verify the actual length estimates of the coastline.

The algorithm starts with obtaining the high resolution spacial coastline map of Australia (see Fig. 2d) from GADM. We then remove small islands and coastline of Tasmania using a ‘*R*’ program and cover the coastline with gridlayer using vector research tools in QGIS. The algorithm counts the number of boxes (*N*) for a specified grid size (*r*) and finally store the output data for calculations. We have used total 17 grid sizes using the measurement scales: 1000, 800, 750, 600, 500, 400, 300, 250, 200, 100, 75, 50, 25, 10, 5, 1, and 0.5 km.

**Figure 5:**
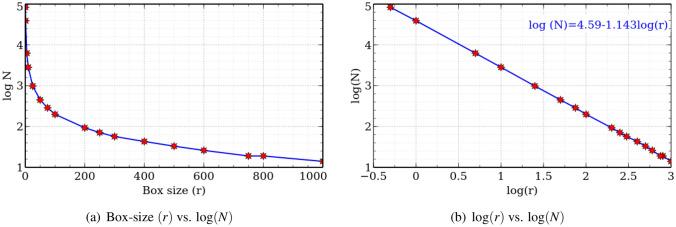
Plots for fractal dimension of coastline of Australia: box-counting method (created using Veusz software, version 3.2.1 available at https://veusz.github.io/download/).

Figure 5 depicts plots of the data inside the scaling regions. In Fig. 5a, box size (*r*) is potted against number of boxes (*N*) (on $\log$ scale). It is evident from the plot that the number of boxes increases rapidly as the size of the measuring scale decreases below 1. This confirms the inverse power law relation between number of boxes and the measurement scale. The $\log N-\log r$ curve is plotted in Fig. 5b, which allow us to determine the fractal dimension by measuring the slope of the best fit line to the computed data $\log (N)=-1.143\log (r)+4.59$ (obtained using the method of least-squares). From this, we get the box-counting dimension of the coastline of Australia as 1.143 which is in close agreement with the divider dimension 1.130 obtained by Richardson^35^, Mandelbrot^28^ having an absolute error of 0.013 and a relative error of just $1.15\%$.

Now using the box-size (*r*), and the equation $L(r)=k\times r^{1-D}$ with $\log (k)$ given as the intercept of $\log N-\log r$, we recover the power law (by fitting the power regression curve) for the coastline length of Australia as:

10 $$\begin{aligned} \displaystyle {L(r)}=38910.4251\, r^{-0.1426} \end{aligned}$$

**Figure 6:**
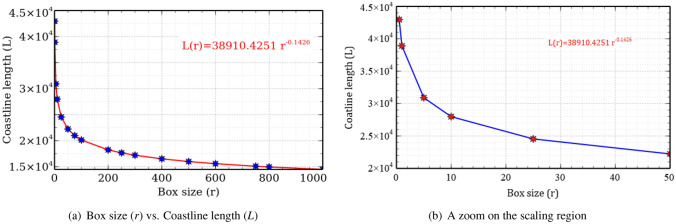
Power law for coastline length of Australia (created using Veusz software, version 3.2.1 available at https://veusz.github.io/download/).

Here, the exponent of *r* is $1-D$. The plot for this is given in Fig. 6a. Note that the length of the coastline increases rapidly as the measurement scale decreases within the scaling regions. In practice, the smallest possible measurement scale within the scaling regions should be selected to calculate the length of the coastline after the scaling regions and fractal dimensions are determined since the length of the coastline increases rapidly with decrease in the measurement scale within the scaling region (see Fig. 6). Therefore, the lower limit of the scaling region is a natural choice for calculating the coastline length. In Fig. 6b, a zoom on the scaling region is shown. From this, we found that the lower limit of scaling region for the coastline of Australia is (nearly) 1 km. Thus, the length of the mainland coastline is approximately 38910 km which is in close agreement with the actual length of 35821 km reported at the beginning of this Section.

As the grid size decreases, the number of features or grid cells increases, as well as memory requirement increases which will slow down the performance of the algorithm. At very small grid sizes, the parallelization is still very challenging and yet to be achieved specially when the coastline (e.g. Australia, Russia, Canada etc.) or the Euclidean object under consideration are very large in size. In all such instances, a truly parallel distributed memory algorithm (via use of MPI, Open MP for inter processor communication) seems to be the only option that can speed up the computations very fast. Another possibility to accelerate calculations is to exploit the massively parallel processing power of the GPUs (graphics processing units) together with CUDA or OpenCL parallel computing platforms.

## Conclusions

Australia has 7th largest coastline in the world and computing its box-counting dimension is challenging particularly with reference to the size of the problem at small scales. In this article, a novel multicore parallel processing algorithm is presented for box dimension calculations of coastlines and other irregular Euclidean objects. The algorithm is successfully applied to investigate the fractal dimension of coastline of Australia.

The box-counting dimension of the coastline of Australia is found to be 1.143 which is close to the existing divider dimension 1.130 computed by Mandelbrot^28^. This confirms the accuracy of the algorithm. We obtained the power law relation for the coastline length using the power regression model (in which the response variable is proportional to the explanatory variable raised to a power) and the computed length of the coastline from the power law produced very good estimate of the actual coastline length within the scaling regions.

The algorithm presented here has many advantages over existing algorithms. The algorithm is generic, scalable and addresses the time-consuming problem of box-dimension calculation for large natural (Euclidean) objects having irregular geometries. It combines fractal geometry with the flexibility of QGIS along with the parallel architecture of the algorithm.

The algorithm can be applied to computing fractal dimension of any coastline and other natural objects such as mountains, rivers, glaciers, natural satellites and even bigger planets. In future we plan to determine the fractal dimension of the largest coastlines e.g. Canada, Indonesia etc. (no results exist till now) by modifying the algorithm into a massively parallel algorithm using message passing interface (MPI) and/or GPU paradigm for inter-processor communications to effectively control the problem size at smaller scales. We remark that the development of CPU, GPU based parallel algorithms in fractal geometry is still in nascent stages and the multi-processing algorithm presented in this article in a small step in this direction. Future will certainly witness a remarkable growth in the design and implementation of such algorithms.
